# Editorial: Metabolome in gastrointestinal cancer

**DOI:** 10.3389/fonc.2023.1234242

**Published:** 2023-06-26

**Authors:** Ciro Isidoro, Yong Teng

**Affiliations:** ^1^ Dipartimento di Scienze della Salute, Università del Piemonte Orientale “A. Avogadro”, Novara, Italy; ^2^ Department of Hematology and Medical Oncology, Winship Cancer Institute, Emory University School of Medicine, Atlanta, GA, United States; ^3^ Wallace H. Coulter Department of Biomedical Engineering, Georgia Institute of Technology & Emory University, Atlanta, GA, United States

**Keywords:** metabolome, gastrointestinal cancers, targeting strategies, cancer metabolism, biomarkers, personalized medicine

## Introduction

Gastrointestinal (GI) cancers, which include the cancer of organs and glandules of the digestive tract such as the esophagus, stomach, intestine, liver, bile ducts and pancreas, are one of the leading causes of cancer deaths worldwide, accounting for nearly 780,000 deaths a year (1). Lifestyle factors, such as smoking, alcohol abuse, and unhealthy diet, along with family genetic predisposition, play a role in many GI cancer types (2, 3). Bacteria, virus and parasitic infections and the gut microbiota also play a role, and certain metabolites have been shown to affect infection, the immune response, and the composition of the gut microbiota (2, 4, 5). This points to the relevance of the metabolism in GI development and the importance of profiling the GI metabolome to understand the mechanisms underlying cancerogenesis and, even more, the progression (6, 7). Metabolites are proven to play an increasingly versatile role in biological interactions, not only as substrates and products of enzyme-driven reactions but also as crucial system regulators (8). Cancer cells can repurpose these systems to drive unwanted proliferation, survival, and growth in cancer, and they can alter their metabolism during progression because of the dynamic interaction with the tumor microenvironment and the availability of metabolic factors such as glucose, amino acids and oxygen (9). This Research Topic collects original and review articles that provide an update on the current knowledge of the metabolomic signature associated with GI cancers, the interactions between metabolites and the GI tumor microenvironment including the microbiota, and the potential of metabolomic studies to develop predictive models and identify biomarkers of GI cancer that could serve for monitoring the progression and, possibly, as targets for a personalized therapy.

## The Research Topic at glance

Cancer cells have a distinct energy metabolism that is different from that of normal, healthy cells. The unique metabolic profile of pancreatic cancer cells has led researchers to investigate the potential of targeting these pathways as a strategy for treating the disease. Chen et al. obtained 22 metabolism-related genes (MRGs) from the MSigDB and gene sequence data in TCGA databases, and ultimately established the prognostic signature for pancreatic cancer based on these MRGs. They highlight that NT5E, a gene encoding the cell surface enzyme CD73, which is involved in the production of extracellular adenosine, is critical for acquiring more aggressive properties of pancreatic cancer by altering its metabolic pattern. Their study could be used to guide survival risk stratification of pancreatic cancer for more precise management of patients.

Esophageal squamous cell carcinoma (ESCC) is a type of cancer that arises from the cells lining the esophagus. One of the major challenges in the early detection of ESCC is the absence of specific symptoms or biomarkers. Symptoms of the disease may not appear until the cancer has progressed to an advanced stage, which can limit treatment options and reduce the chances of survival. Wang et al.‘s group performed UPLC-MS/MS on 450 ESCC patients and 588 controls, consisting of a discovery group and two validation groups, to identify biomarkers for early detection and prognosis. Through metabolite profiling, they found that dysregulated amino acids and lipid metabolism are critical metabolic signatures of ESCC and targeting the retinol and linoleic acid pathways may be a way to develop new mechanism-based therapeutic approaches. Their work provides novel insights for early detection and risk stratification to advance treatment options that contribute to improved outcomes for ESCC patients.

It’s noteworthy that metabolite profiling of serum and urine can provide insight into the metabolic changes that occur during cancer development and progression. For example, cancer cells often exhibit altered energy metabolism, including increased glycolysis and altered amino acid metabolism. These metabolic changes can result in the production and release of specific metabolites that can be detected in serum and urine. Based on this concept, Ouyang et al. collected urine and serum samples from 70 paired healthy and ESCC patients and investigated their changes using high-resolution 600 MHz 1H NMR. The paralleled patient-matched metabolites of ESCC cancer tissue and corresponding distant non-cancerous mucosa were also studied and used as references to determine biofluid metabolic biomarkers. This study not only provides evidence that ^1^H-NMR-based metabolomics is a simple, efficient and inexpensive technique for detecting cancer-associated metabolic fingerprints, but also established an optimized metabolic profile to improve the non-invasive diagnosis of ESCC by combining serum and urine biomarkers. However, due to the small sample size, the limited number of precancerous lesions and tumors at each stage, and the lack of genomics-derived molecular features, validation of the metabolic pathway disturbances identified in these ESCC patients by other independent ESCC patient cohorts is warranted.

The human gut microbiota, composed of trillions of microorganisms, plays a critical role in human health and disease, including colorectal cancer (CRC). Metabolic interactions between the host and the microbiota are thought to be key drivers of CRC development and progression. Gut microbiota-targeted interventions, such as probiotics, prebiotics, and dietary interventions, have shown promise in modulating gut microbiota composition and function and reducing CRC risk. However, more research is needed to fully understand the complex interplay between the gut microbiota and CRC development and progression, and to develop effective microbiota-targeted interventions for CRC prevention and treatment. Li et al.‘s review summarized the recent research progress on the alterations of gut flora and its metabolites associated with CRC. They also discussed the application of association analysis of metabolomics and gut microbiome in the diagnosis, prevention and treatment of CRC. Most importantly, they also provide the perspectives of the problems and development direction of this association analysis in the study of CRC. The delineation of how gut microbiota and their metabolites influence the progression and causation of CRC, and the association analysis of metabolomics and gut microbiome will allow us to shed light on the steps required for the prevention, diagnosis, and therapy of CRC.

## Conclusion and perspectives

There is a clear need to better understand the pathogenesis and pathophysiology of GI cancers, to identify and validate new molecular determinants for GI cancer management, and to develop new therapeutic options for patients with GI malignancies. Over the past decade, the role of the metabolome in GI malignancies has begun to emerge. The metabolome is the global collection of all low molecular weight metabolites that are produced by cells during metabolism and provides a direct functional readout of cellular activity and physiological status. Metabolomics is a high-throughput analytical strategy to qualify or quantify as many metabolites as possible in the metabolome and recent analyses in this area have yielded useful results in the treatment and management of GI cancers. Our understanding of metabolome in GI malignancies can be employed to identify biomarkers for prediction, diagnosis, and prognosis, and this will positively reflect in personalized targeted therapy. Therefore, expanding the knowledge base in the metabolome will have a tremendous impact on the diagnosis, management, and treatment of GI cancers.

## Author contributions

YT and CI wrote, revised, and edited the editorial. All authors contributed to the article and approved the submitted version.
